# Longitudinal evaluation of recurrent thyrotoxicosis in a single patient across four pregnancies: a case report and literature review

**DOI:** 10.1530/EDM-25-0175

**Published:** 2026-06-15

**Authors:** Yosuke Kaido, Hiroto Minamino, Hidefumi Inaba, Daisuke Kosugi, Gen Inoue, Daisuke Yabe

**Affiliations:** ^1^ Department of Diabetes, Endocrinology, and Nutrition, Kyoto University Graduate School of Medicine, Kyoto, Japan; ^2^ Department of Diabetes and Endocrinology, Wakayama Red Cross Hospital, Wakayama, Japan; ^3^ Department of Physiology, Wakayama Medical University Faculty of Medicine, Wakayama, Japan; ^4^ Wakayama Blood Center, Wakayama, Japan

**Keywords:** gestational transient thyrotoxicosis, Graves’ disease, painless thyroiditis, recurrent thyrotoxicosis

## Abstract

**Summary:**

We report the case of a 37-year-old woman who presented with hyperemesis gravidarum at 11 weeks of gestation. Laboratory examination revealed severe thyrotoxicosis (TSH: <0.005 μIU/mL, FT3: 12.95 pg/mL, FT4: 3.93 ng/dL) with negative anti-TSH receptor antibody, while serum human chorionic gonadotropin (hCG) was markedly elevated at 198,983 mIU/mL. She was diagnosed with gestational transient thyrotoxicosis (GTT), although the presence of thyroid-stimulating antibody raised suspicion for concomitant Graves’ disease (GD). Because of severe and clinically burdensome thyrotoxic symptoms, including excessive sweating and weight loss, propylthiouracil therapy was initiated, but thyrotoxicosis recurred after 20 weeks of gestation alongside elevated serum hCG levels, prompting a switch to thiamazole. Genetic testing revealed no TSHR mutations. Postpartum, both hCG and thyroid hormone levels normalized without treatment. During the second pregnancy, she experienced a miscarriage at nine weeks. During the third pregnancy, an elective abortion was performed due to a fetal genetic disorder. Both pregnancies were marked by severe thyrotoxicosis, consistent with GTT. During the fourth pregnancy, at 11 weeks, she developed hyperemesis gravidarum and was diagnosed with GTT. The persistently elevated thyroglobulin levels (>200 ng/mL; reference range:, <33.7 ng/mL) were also compatible with a possible contribution from painless thyroiditis (PT). Serum hCG levels normalized after the second trimester. Recurrent and sustained thyrotoxicosis during pregnancy across multiple pregnancies in the same patient is rare and requires careful differentiation among possible etiologies, including GTT, GD, and PT. This case underscores the importance of comprehensive monitoring of placental function, thyroid autoimmunity, and thyroiditis throughout pregnancy to ensure accurate diagnosis and tailored management for both mother and fetus.

**Learning points:**

- The diagnosis of recurrent thyrotoxicosis in pregnancy is complex, as distinct entities can overlap clinically.
- Comprehensive phenotyping – incorporating thyroid autoantibodies, serum hCG, Tg, and ultrasound – guides diagnosis; reserve TSHR testing for atypical or persistent cases.
- Careful longitudinal monitoring across pregnancies enables optimized maternal–fetal outcomes.

## Background

Thyroid function is closely associated with pregnancy (1). Thyroid hormones are crucial for fetal development, particularly in neurodevelopment during the first trimester when the fetus depends entirely on maternal thyroid hormone supply (1). Maternal thyroid dysfunction is associated with adverse obstetric outcomes, and untreated hyperthyroidism increases miscarriage and preterm birth risk (2). Therefore, the appropriate management of thyroid disorders throughout pregnancy is essential for maternal–fetal health.

Graves’ disease (GD) and gestational transient thyrotoxicosis (GTT) are the two most common causes of hyperthyroidism during pregnancy (1). GD is an autoimmune disorder characterized by anti-thyroid-stimulating hormone (TSH) receptor antibodies, leading to hyperthyroidism. In contrast, GTT is defined as transient thyrotoxicosis caused by the thyroid-stimulating effect of human chorionic gonadotropin (hCG), a hormone that temporarily suppresses TSH and elevates free thyroid hormone levels during early pregnancy (3). Serum hCG levels typically peak at 10–12 weeks of pregnancy, coinciding with the nadir of TSH levels. GTT typically resolves as hCG levels decline after the first trimester (3). Considering the distinct pathophysiology, clinical course, and management strategies of GD, GTT, and other forms of thyrotoxicosis, accurate diagnosis is essential.

Here, we report the case of a 37-year-old woman who developed multiple forms of thyrotoxicosis including GTT, persistent thyrotoxicosis into late pregnancy accompanied by sustained hCG elevation, and possibly GD or painless thyroiditis (PT) across four singleton pregnancies.

## Case presentation

A 37-year-old Japanese woman with no family history of autoimmune thyroid disease presented at 11 weeks of gestation during her first pregnancy, complaining of severe nausea and vomiting, accompanied by excessive sweating and weight loss. Her medical history was unremarkable. Her height was 167 cm, body weight was 46 kg, and heart rate was 99 bpm, with other vital signs within normal ranges. Physical examination revealed diffuse thyroid enlargement without ophthalmopathy.

## Investigation

**Figure 1:**
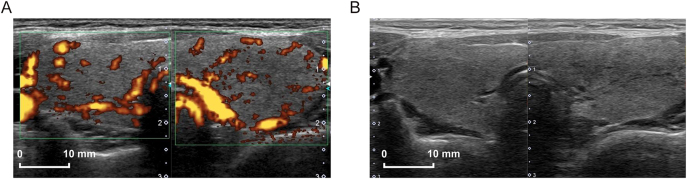
Representative thyroid ultrasound images. (A) Doppler ultrasound at 11 weeks of gestation during the first pregnancy shows diffuse thyroid enlargement with increased vascularity. The right lobe measured 22.1 × 15.9 mm, and the left lobe measured 25.3 × 16.8 mm. (B) Thyroid ultrasound at nine weeks of gestation during the fourth pregnancy demonstrates a heterogeneous parenchyma without significantly increased blood flow. The right lobe measured 21.6 × 15.7 mm, and the left lobe measured 25.7 × 16.0 mm. The scale bar represents 10 mm.

Laboratory examination at 11 weeks of gestation indicated thyrotoxicosis with free triiodothyronine (FT3): 12.95 pg/mL; reference range: 2.30–4.00 pg/mL, free thyroxine (FT4): 3.93 ng/dL; 0.90–1.70 ng/dL, TSH: < 0.01 µIU/mL; 0.50–5.00 µIU/mL, with markedly elevated hCG level of 198,983 mIU/mL (Table 1). GTT was initially suspected; however, third-generation anti-TSH receptor antibody (TRAb) was undetectable (<0.3 IU/L), while TSH receptor-stimulating antibody (TSAb), measured using the Yamasa TSAb enzyme immunoassay (EIA) kit, was weakly positive at 128% (reference range: < 120%) (Table 1). Anti-thyroglobulin antibody (TgAb) and anti-thyroid peroxidase antibody (TPOAb) were negative (Table 1). Thyroid ultrasound further showed diffuse gland enlargement with increased vascularity (Fig. 1A). Sanger sequencing of exons 1–10 of the TSH receptor gene revealed no sequence variants, including no rare variants or previously reported variants associated with increased hCG sensitivity, such as substitutions at lysine 183 to asparagine or arginine (4, 5). The primer sequences of ten exons of the TSH receptor gene and PCR conditions were designed with reference to the method described by de Roux *et al.* (6). Genetic analysis was approved by the Ethics Committee of Wakayama Medical University, and written informed consent for genetic testing was obtained.

**Table 1:** Laboratory results of four pregnancies. Abnormal values are shown in bold.

	1st (11 weeks)	2nd (9 weeks)	3rd (13 weeks)	4th (9 weeks)	Reference ranges
TSH, μIU/mL	**<0.01**	**<0.005**	**<0.005**	**<0.005**	0.50–5.00
FT3, pmol/L	**19.89**	**6.77**	**15.11**	**9.34**	3.53–6.14
FT4, pmol/L	**50.58**	**25.35**	**44.66**	**30.37**	11.6–21.9
FT3/FT4 ratio	3.30	2.24	2.84	2.58	
Tg, ng/mL	**128**	ND	ND	**344**	<33.70
TRAb, IU/L	<0.8	ND	ND	<0.8	<2.0
TSAb, %	**128**	ND	ND	98	<120
TgAb, IU/mL	10	ND	ND	<3.00	<28.0
TPOAb, IU/mL	2.4	ND	ND	<3.00	<16.0
hCG, mIU/mL	198,983	ND	201,214	168,634	N/A

TSH, thyroid-stimulating hormone; FT3, free triiodothyronine; FT4, free thyroxine; Tg, thyroglobulin; TRAb, anti-TSH receptor antibody; TSAb, TSH receptor-stimulating antibody; TgAb, anti-thyroglobulin antibody; TPOAb, anti-thyroid peroxidase antibody; hCG, human chorionic gonadotropin. ND, not determined. N/A, not applicable.

## Treatment

**Figure 2:**
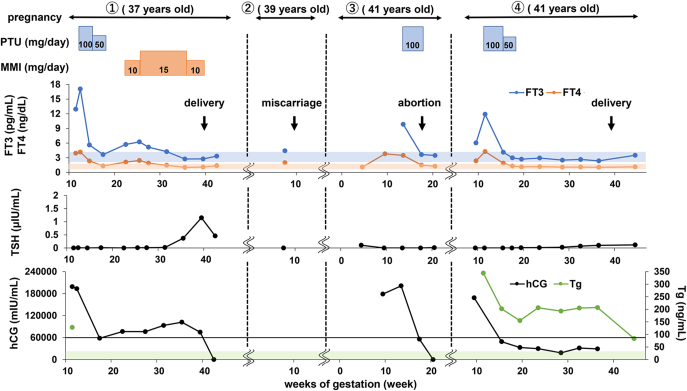
Clinical course of the four pregnancies. In the first pregnancy at age 37, PTU was started at 12 weeks, normalizing thyroid hormones, but persistent hCG elevation led to MMI initiation at 22 weeks. Thyroid function remained stable until delivery at 39 weeks. The second and third pregnancies followed typical GTT courses, ending in miscarriage and elective termination. In the fourth pregnancy, PTU started at 11 weeks normalized thyroid function by 17 weeks, but persistent thyroglobulin elevation and TSH suppression suggested painless thyroiditis. A healthy male was delivered at 39 weeks and 6 days. The normal FT3, FT4, and Tg ranges are indicated in blue, orange, and green, respectively. FT3, free triiodothyronine; FT4, free thyroxine; GTT, gestational transient thyrotoxicosis; hCG, human chorionic gonadotropin; MMI, thiamazole; PT, painless thyroiditis; PTU, propylthiouracil; Tg, thyroglobulin.

Considering the possibility of coexisting GD and her marked thyrotoxic symptoms, including profound nausea, vomiting, excessive sweating, weight loss, and severe hyperemesis, propylthiouracil (PTU: 100 mg/day) was initiated at 12 weeks rather than expectant management (Fig. 2). As hCG levels declined, thyroid function improved, and PTU was discontinued at 17 weeks. However, the hCG levels increased again in the second and third trimesters, resulting in recurrent thyrotoxicosis. Thiamazole (MMI: 10 mg/day) was started at 22 weeks and stabilized thyroid function until delivery (Fig. 2).

## Outcome and follow-up

At 39 weeks and 1 day, she delivered a female neonate (2,850 g) with normal thyroid function. Both the patient and the neonate had an uncomplicated perinatal course, and the patient’s thyroid function normalized postpartum. TRAb reassessed at 4 months postpartum was negative (<0.8 IU/L).

At age 39, she became pregnant with her second child and developed hyperemesis and thyrotoxicosis with FT3: 4.41 pg/mL, FT4: 1.97 ng/dL, and TSH: <0.005 μIU/mL. She miscarried at 9 weeks of gestation.

Two years later, at age 41, she conceived for the third time via frozen embryo transfer. At 10 weeks of gestation, she again presented with thyrotoxicosis with FT4: 3.80 ng/dL and TSH: <0.005 μIU/mL, accompanied by a markedly elevated hCG level of 178,793 mIU/mL and hyperemesis gravidarum. Due to the severity of symptoms, PTU 100 mg/day was initiated. The pregnancy was terminated at 17 weeks due to fetal genetic abnormalities. Following terminatiuon, thyroid function spontaneously normalized, consistent with the clinical course of GTT.

At 41 years of age, she conceived her fourth pregnancy via frozen embryo transfer, 8 months after the abortion of her third pregnancy. At 9 weeks of gestation, she presented with severe nausea, vomiting, and thyrotoxicosis with FT3: 6.08 pg/mL, FT4: 2.36 ng/dL, and TSH:L <0.005 μIU/mL accompanied by elevated hCG levels of 168,634 mIU/mL. Thyroid autoantibodies, including TSAb, were negative, while thyroglobulin (Tg) levels were markedly elevated at 344 ng/mL (reference range: <33.70 ng/mL) (Table 1). Thyroid ultrasound examination revealed heterogeneous parenchyma with focal hypoechoic areas, without increased vascularity (Fig. 1B). Two weeks later, FT3 and FT4 levels rose further, and her hyperemesis worsened. PTU 100 mg/day was initiated at 11 weeks of gestation. Unlike the first pregnancy, hCG levels normalized during the second trimester, resulting in normalization of FT3 and FT4, and PTU was discontinued at 17 weeks, consistent with the typical course of GTT. However, as shown in Fig. 2, TSH remained suppressed and Tg remained elevated until delivery, exceeding 100 ng/mL from 15 to 36 weeks, raising the possibility of concomitant PT. Thyroid function remained stable without further intervention, and at 39 weeks and 6 days, she delivered a male neonate (3,030 g) with normal thyroid function. Both the patient and the neonate had an uncomplicated perinatal course, and her thyroid function spontaneously normalized postpartum.

## Discussion

We report a rare case of recurrent pregnancy-associated thyrotoxicosis across four pregnancies, most consistent with GTT overall but with possible contributions from GD in the first pregnancy and PT in the fourth pregnancy (Table 2). The first pregnancy was initially diagnosed as GTT based on elevated hCG levels; however, weakly positive TSAb and increased thyroid blood flow on ultrasound were compatible with possible coexisting GD. Moreover, thyrotoxicosis persisted due to sustained elevation of hCG beyond the first trimester. The second and third pregnancies, which did not progress to delivery, followed a clinical course consistent with GTT. During the fourth pregnancy, PT was considered a possible diagnosis due to persistently suppressed TSH levels, elevated Tg levels (>100 ng/mL) until delivery, and a heterogeneous echotexture observed on thyroid ultrasonography (Table 2 and Fig. 2). Notably, thyrotoxicosis persisted into late pregnancy during both the first and fourth pregnancies, both of which resulted in successful deliveries. Several cases of recurrent gestational thyrotoxicosis have been reported, each with distinct clinical features (Table 3) (4, 7, 8). Unlike previous reports limited to GTT and lacking data on hCG levels or TSH receptor (TSHR) mutations, our case uniquely demonstrated recurrent thyrotoxicosis from multiple etiologies across four pregnancies. Comprehensive evaluation of serum hCG and TSHR mutation status helped narrow the differential diagnosis and informed individualized management, although the etiologic classification remained probabilistic.

**Table 2:** Summary of four pregnancies. Abnormal values are shown in bold.

	1st pregnancy	2nd pregnancy	3rd pregnancy	4th pregnancy
At age, years	**37**	**39**	**41**	**41**
Diagnosis				
GTT	**✓**	**✓**	**✓**	**✓**
GD(possible)	**✓**	-	-	-
PT (possible)	-	-	**-**	**✓**
Hyperemesis	**++**	**+**	**+**	**+**
FT3/FT4 ratio	**3.30**	2.24	2.84	2.58
TRAb	Negative	ND	ND	Negative
TSAb	**Positive**	ND	ND	Negative
Tg, ng/mL	**128**	ND	ND	**344**
Thyroid echography	Diffuse enlargement/increased blood flow	ND	ND	Heterogeneous parenchyma/no increased blood flow
Outcome	Delivery (at 39 weeks); female: 2,850 g	Miscarriage (at 9 weeks)	Abortion (at 17 weeks)	Delivery (at 39 weeks); male: 3,030 g

GTT, gestational transient thyrotoxicosis; GD, Graves’ disease; PT, painless thyroiditis; FT3, free triiodothyronine; FT4, free thyroxine; TRAb, TSH receptor antibody; TSAb, TSH receptor-stimulating antibody; Tg, thyroglobulin.

✓, likely; -, less likely; ND, not determined.

**Table 3:** Literature review of recurrent gestational thyrotoxicosis.

Study	Number of pregnancies	Age, years	Diagnosis	Therapy	Outcome	Serum hCG levels	TSHR mutation
Navaneethakrishnan *et al.* (7)	2		GTT	PTU	Delivery	Not tested	Not tested
	1st	25					
	2nd	26					
Navaneethakrishnan *et al.* (7)	5						Not tested
	1st	ND	ND	None	Delivery	Not tested	
	2nd/3rd	ND	ND	None	Miscarriage	Not tested	
	4th	29	GTT	CMZ	Delivery	Not tested	
	5th	31					
Rodien *et al.* (4)	4						Positive (Lys183Arg)
	1st/2nd	ND	ND	None	Miscarriage	Not tested	
	3rd	27	GTT	PTU	Delivery	Within normal range	
	4th	29					
Jeffcoate *et al.* (8)	2						Not tested
	1st	26	GTT	CMZ	Delivery	Within normal range	
	2nd	28	GTT	CMZ	Delivery	Elevated early➝normalized	
This case	4						No pathogenic TSHR variants detected
	1st	37	GTT + GD (possible)	PTU➝MMI	Delivery	Elevated early➝sustained high	
	2nd	39	GTT	None	Miscarriage	Not tested	
	3rd	41	GTT	PTU	Abortion	Elevated early	
	4th	41	GTT + PT (possible)	PTU	Delivery	Elevated early➝normalized	

GTT, gestational transient thyrotoxicosis; GD, Graves’ disease; PT, painless thyroiditis; PTU, propylthiouracil; MMI, methimazole; CMZ, carbimazole; hCG, human chorionic gonadotropin; TSHR, thyroid-stimulating hormone receptor.

ND, not determined (information not available in the original report).

GD and GTT are the most common causes of hyperthyroidism during pregnancy, though their pathophysiology and clinical course differ significantly. GD is an autoimmune disorder driven by TSAb, which leads to persistent thyrotoxicosis. In contrast, GTT is caused by hCG-mediated stimulation of the TSH receptor (2). During pregnancy, immune modulation generally suppresses autoimmune activity, leading to an improvement in GD, with hyperthyroidism frequently ameliorating during the second and third trimesters (9). Conversely, GTT is a transient condition, as hCG levels typically peak at 10–12 weeks of gestation and subsequently decline (3). Although most GTT cases resolve spontaneously, prolonged hCG elevation, such as in multiple pregnancy or in association with gestational diabetes, can result in persistent thyrotoxicosis (3).

Differentiating between new-onset GD and GTT is critical, particularly in cases like ours, where both conditions can coexist. Yoshihara *et al.* reported that the FT3/FT4 ratio was significantly higher in GD (3.03 vs 2.32), with a cutoff value of 2.70 yielding 77% sensitivity and 88% specificity for distinguishing between the two conditions (10). Furthermore, GD is characterized by the presence of TSAb or TRAb, whereas these antibodies are absent in GTT. During the first pregnancy, a higher FT3/FT4 ratio (3.30), weakly positive TSAb, and increased thyroid blood flow were compatible with possible coexisting GD. However, some aspects of the clinical presentation were atypical for GD, as only TSAb was weakly positive and thyroid function improved rapidly following the initiation of PTU. The weakly positive TSAb result may have been a false-positive finding secondary to markedly elevated hCG levels. Therefore, the first pregnancy was considered most consistent with hCG-induced thyrotoxicosis, although coexisting GD could not be excluded.

Other causes of thyrotoxicosis include toxic nodules and PT, which typically present in the first postpartum year (9). However, PT can also occur after pregnancy loss, including abortion, likely via immune reactivation (9). In our case, the third pregnancy ended in induced abortion, and the fourth pregnancy occurred eight months later. Although this interval cannot be regarded as short, the possibility of coexisting PT cannot be completely excluded because several findings during the fourth pregnancy were not entirely typical of uncomplicated GTT, including negative TSAb, persistent TSH suppression after the decline in hCG, serum thyroglobulin (Tg) levels >200 ng/mL throughout gestation, and heterogeneous echotexture without increased vascularity on ultrasonography (11). Notably, Tg >100 ng/mL is uncommon even in normal pregnancy, occurring in 3% before 16 weeks and 5% at 32 weeks of gestation (9). However, prolonged Tg elevation in this case may also be explained within the spectrum of hCG-driven thyroid stimulation, particularly because the Tg level during the first pregnancy was already elevated (128 ng/mL). Therefore, in the absence of serial ultrasonographic changes over time or evidence of a subsequent hypothyroid phase requiring levothyroxine replacement, PT cannot be confidently established as a definitive diagnosis. Taken together, the thyrotoxicosis in the fourth pregnancy was most consistent with GTT, with PT remaining a possible contributing factor.

In practice, treatment decisions for hyperthyroidism during pregnancy are guided not only by the presumed etiology but also by the severity and persistence of thyrotoxicosis. GTT is typically transient and self-limiting, and anti-thyroid drugs (ATDs) are generally not required (3). In contrast, GD necessitates treatment to reduce risks such as preeclampsia, low birth weight, miscarriage, and preterm delivery (2). During the first pregnancy, PTU was started at 12 weeks for suspected GD but was discontinued at 17 weeks. However, thyrotoxicosis recurred at 22 weeks, prompting the initiation of MMI therapy. Although GTT rarely requires ATD therapy, treatment may be warranted in selected cases with severe or persistent thyrotoxicosis (1, 2, 3, 4, 5, 11). Specific TSH receptor mutations that enhance sensitivity to hCG, particularly codon 183 substitutions such as Lys183Asn and Lys183Arg in the extracellular ligand-binding domain, have been implicated in sustained gestational thyrotoxicosis and may respond well to PTU therapy (4, 5). In our case, Sanger sequencing of TSHR exons 1–10 revealed no pathogenic variants; nevertheless, ATD therapy was required and proved effective because the thyrotoxicosis was clinically severe and persisted into late pregnancy.

Given the diverse etiologies of hyperthyroidism during pregnancy, careful evaluation of hCG levels, TRAb, Tg, imaging findings, and genetic factors is important, although precise etiologic classification may remain probabilistic. This case has several limitations. First, TSAb was not remeasured in the late course of the first pregnancy, while TRAb reassessed postpartum was negative. Second, follow-up ultrasonography was not performed after the second trimester to assess whether PT persisted after hCG levels had declined. Third, TSAb was measured using the Yamasa TSAb enzyme immunoassay (EIA), which incorporates anti-TSH antibodies to reduce endogenous TSH interference. Although this assay differs from the recently reported homogeneous bioassay, interference testing in that methodological study showed only a minor effect of hCG at 25,000 mIU/mL (4.1% change in TSAb activity) (12). Because the hCG concentration in our patient was substantially higher, assay-related influence cannot be completely excluded.

In conclusion, this case represents recurrent pregnancy-associated thyrotoxicosis across multiple pregnancies, most consistent with GTT overall, with possible contributions from GD in the first pregnancy and PT in the fourth pregnancy. It underscores the importance of careful longitudinal evaluation and cautious etiologic interpretation in guiding management.

## Declaration of interest

The authors declare that there is no conflict of interest that could be perceived as prejudicing the impartiality of the work reported.

## Funding

This research did not receive any specific grant from any funding agency in the public, commercial, or not-for-profit sector.

## Patient consent

Written informed consent for publication of their clinical details and clinical images was obtained from the patient.

## Author contribution statement

All authors made individual contributions to authorship. YK, HM, and HI drafted the manuscript. YK, HM, HI, and GI were involved in the diagnosis and management of the patient. HM, HI, DK, and DY critically revised the manuscript for intellectual content. All authors reviewed and approved the final draft.
